# The HAART cell phone adherence trial (WelTel Kenya1): a randomized controlled trial protocol

**DOI:** 10.1186/1745-6215-10-87

**Published:** 2009-09-22

**Authors:** Richard T Lester, Edward J Mills, Antony Kariri, Paul Ritvo, Michael Chung, William Jack, James Habyarimana, Sarah Karanja, Samson Barasa, Rosemary Nguti, Benson Estambale, Elizabeth Ngugi, T Blake Ball, Lehana Thabane, Joshua Kimani, Lawrence Gelmon, Marta Ackers, Francis A Plummer

**Affiliations:** 1 Department of Medical Microbiology, University of Nairobi, UNITID Building, Nairobi, Kenya; 2 Department of Medical Microbiology, University of Manitoba, Health Sciences Centre, Winnipeg, Manitoba, Canada; 3 BC Centre for Excellence in HIV, St. Paul's Hospital, University of British Columbia, Vancouver, BC, Canada; 4 School of Kinesiology and Health Sciences, Department of Psychology, York University, York, Ontario, Canada; 5 Department of Economics, Georgetown University, Washington, USA; 6 Division of Allergy and Infectious Diseases, Department of Medicine, University of Washington, Seattle, WA, USA; 7 University of Nairobi Institute of Tropical and Infectious Diseases (UNITID), Nairobi, Kenya; 8 Department of Clinical Epidemiology & Biostatistics, McMaster University, Hamilton, Ontario, Canada; 9 US Centers for Disease Control and Prevention (CDC), Nairobi, Kenya; 10 National Microbiology Laboratory, Public Health Agency of Canada, Winnipeg, Manitoba, Canada

## Abstract

**Background:**

The objectives are to compare the effectiveness of cell phone-supported SMS messaging to standard care on adherence, quality of life, retention, and mortality in a population receiving antiretroviral therapy (ART) in Nairobi, Kenya.

**Methods and Design:**

A multi-site randomized controlled open-label trial. A central randomization centre provided opaque envelopes to allocate treatments. Patients initiating ART at three comprehensive care clinics in Kenya will be randomized to receive either a structured weekly SMS ('short message system' or text message) slogan (the intervention) or current standard of care support mechanisms alone (the control). Our hypothesis is that using a structured mobile phone protocol to keep in touch with patients will improve adherence to ART and other patient outcomes. Participants are evaluated at baseline, and then at six and twelve months after initiating ART. The care providers keep a weekly study log of all phone based communications with study participants.

Primary outcomes are self-reported adherence to ART and suppression of HIV viral load at twelve months scheduled follow-up. Secondary outcomes are improvements in health, quality of life, social and economic factors, and retention on ART. Primary analysis is by 'intention-to-treat'. Sensitivity analysis will be used to assess per-protocol effects. Analysis of covariates will be undertaken to determine factors that contribute or deter from expected and determined outcomes.

**Discussion:**

This study protocol tests whether a novel structured mobile phone intervention can positively contribute to ART management in a resource-limited setting.

**Trial Registration:**

Trial Registration Number: NCT00830622

## Background

The most important factor for sustainable treatment of HIV/AIDS for individuals and programs globally is highly consistent use of highly active antiretroviral therapy (ART). For patients, this means adhering to daily or twice daily medication schedules, typically at a minimum of at least 95% of the time, and for programs this means supporting and monitoring patients to achieve those goals.[1] The World Health Organization and UNAIDS have outlined ambitious goals of universal access to those in need among the 28 million people infected with HIV globally, and include a directive to embrace new technologies to help achieve that goal.[2]

Mobile telephones have transformed telephone communications dramatically in resource limited settings. In Kenya, mobile phones penetration has reached over 60% and Africa has the highest rate of new uptake in the world[3]. The reach of cellular networks among HIV infected persons may be even higher than the general African population since both HIV and wireless network coverage are preferenced to areas of higher population densities such as urban areas and transport routes. Currently, mobile phones are used intensely in personal lives and business transactions in the region. We therefore feel that structured mobile phones communications can substantially improve clinical management of HIV patients in resource-limited settings[4].

No previous RCTs of cell-phones for ART adherence have been reported, thus, no systematic review is available. This protocol outlines our proposed methods of a multi-site randomized clinical trial (RCT) that tests if a novel mobile phone communication intervention improves adherence to HAART, and improves other patient outcomes in Kenya. Patient outcomes assessed include mortality, morbidity, quality of life, social and economic indicators. We hypothesize that the cell phone intervention may directly or indirectly improve these outcomes. If the intervention improves HAART adherence (primary outcome) and the drug effect (suppression of plasma viral load), then health, quality of life, social and economic indicators could all be consequently improved. Since the intervention also provides an enhanced communication link between patients and healthcare providers, patient support may also influence patient outcomes directly, independent of drug adherence. By using intention to treat (ITT) analysis, we will broadly assess the effect of the intervention on patient outcomes. By using sensitivity and subgroup analyses, we will assess the intervention effect more specifically.

## Methods and Design

We registered our clinical trial with ClinicalTrials.gov and received the number NCT00830622.

### Funding

Funding was obtained through the President's Emergency Plan for AIDS Relief (PEPFAR) through a cooperative agreement, #U62/PS024510, from the US Centers for Disease Control and Prevention.

### Study design

**Figure 1:**
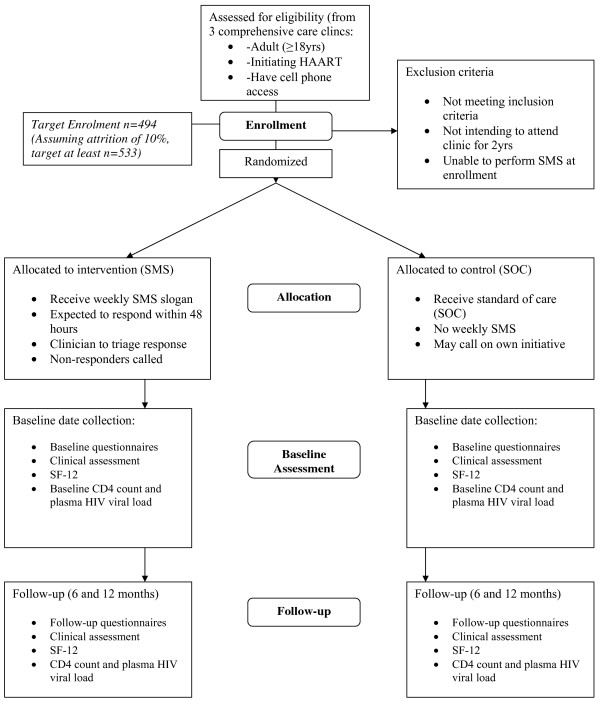
**Consort diagram of study design**.

The study is a multi-site RCT. Patients will be randomized to the cell phone (SMS) intervention group versus the standard of care (SOC) control (no SMS) at a 1:1 allocation ratio (see Figure 1 Consort diagram).

### Randomization

A project statistician generated 1:1 randomization numbers for study arm assignments using a random number generating program. The randomization was stratified in a 2:2:1 ratio to the three clinics (the rural clinic having the lower number of assignments). Written allocation of assignment is sealed in individual opaque envelopes marked with study identification numbers which are distributed to all three study clinics in sufficient quantity to allocate the target numbers of participants. After consenting to participate and meeting inclusion criteria, screened subjects are enrolled and immediately afterward are assigned to the randomized study arm by a clinician opening the sealed envelopes to determine allocation. Age, gender, and CD4 T cell counts will be assessed for balance of study arm allocation at each site.

### Setting and participants

Subjects are recruited from three HIV comprehensive care clinics (CCC) in Kenya. One facility is an urban university-run clinic situated in a lower socioeconomic area of Nairobi, the second is an urban clinic attached to a faith-based hospital situated in a middle income area of Nairobi, and the third is a government-run clinic situated in a vast rural district of Kenya populated by pastoralists.

Patients are eligible to participate if they are initiating HAART for the first time (within two weeks before or after enrolment screening), if they are adults (at least 18 yrs old), if they owned or had sufficient access to a mobile phone (shared access is allowed if access is daily and the phone owner agrees to participate), and if they were able to sufficiently operate a cell phone to communicate using text-messaging (help by a partner is sufficient for illiterate subjects). Patients need to provide oral and written consent to participate. For patients who are illiterate, clear oral consent is obtained. Patients are excluded if they do not meet inclusion criteria or are not planning to attend the enrolment clinic for the planned study period (at least two years). Patients are considered newly initiating HAART if they have not taken HAART in at least one year and have not taken it ever for more than 2 months. Most prevention of mother to child transmission (PMTCT) regimens in Kenya include limited antiretroviral regimens and not true HAART, so participation in PMTCT is not an exclusion criteria. Therefore, the study covers a broad range of patients attending comprehensive care clinics (CCC) for ART in Kenya.

Participants are compensated for their participation time with money for lunch on study visit days (100 Kenya shillings). Patients are not provided cell phones or network airtime credit.

### Intervention

The intervention being evaluated is a structured cell phone initiative to improve communications between healthcare providers and patients. The intervention protocol was conceived in 2005 by several brainstorming meetings between health care provider staff at the clinics, and ultimately incorporated feedback from pilot patients from a survey and from small group sessions[3].

Every week, on Monday mornings, the site-clinician (typically a nurse or clinical officer) sends a text message (SMS) to all subjects in the intervention arm to remind them about their support and enquire into how they are doing. Typically, a slogan is used such as "Mambo?" which is Kiswahili for "How are you?", but similar slogans in other local languages can also be used at the clinicians' discretion. To simplify their work, the clinicians use the 'send to many' function on their cell phone for mass/group messaging. Intervention subjects are instructed to respond within 48 hours either that they are doing well ("Sawa") or that they have a problem ("Shida") and request assistance. Sending an SMS is inexpensive (less than $0.05); however, in some circumstances where network credit is not available or was impractical, the patients can use the free 'flashback' function to trigger a call from the clinician. The clinician then calls to follow-up and provide triage to any subjects that respond that there is a problem, or subjects who fail to respond within two days.

Clinicians are only expected to respond to text messages during regular clinic hours, but are not restricted to do so. Intervention participants are instructed that the intervention is a clinic utility and that all emergencies should be handled through their usual means such as attending the nearest hospital emergency department. All participants receive a brief training from the clinicians on use of the phone and the protocol if they were in the intervention. Use of any of the cell phone network providers is allowed. Receiving SMS text messages or voice calls in Kenya is free on all networks.

### Control

Patients randomized to the control arm receive their usual standard of care (SOC) clinic support but are not sent the weekly SMS slogans from the clinicians. They are, however, free to call the clinic staff at any time on their own initiative. All cell phone communications between clinicians and study participants, in both study arms, are recorded in a study log.

### Objectives

#### Primary objectives

The study is intended to test whether the cell phone intervention (SMS) improves self-reported adherence to HAART and whether it affects a biological surrogate marker of adherence, specifically suppression of plasma HIV RNA load at 12 months follow-up. We hypothesize that the SMS intervention will remind patients taking HAART about clinic support and reinforce regular communications in order to sort out any adherence related problems efficiently. Thus, we hypothesize the intervention will positively support patients' adherence to HAART and consequent suppression of HIV viral load compared to patients who only receive the standard of care (control).

#### Secondary objectives

We will also test the cell phone intervention (SMS) ability to improve health, quality of life, social, economic outcomes and retention in ART programs. We hypothesize that patients who receive the weekly SMS reminders (intervention) may have improved immune reconstitution (CD4 count), increased body weight, fewer opportunistic infections, fewer adverse health events, improved quality of life, delayed death, improved social factors such as disclosure of their HIV status, and improved economic indicators compared to those that do not receive the intervention (control). We also hypothesize that subjects in the intervention arm will be more frequently retained in the ART program.

### Outcome measures

#### Sampling and enrolment

We determined that continuous sampling was impractical since the clinics are often extremely busy and clinic staff are obliged to provide rapid patient care in order to meet service targets. Only a few patients (typically up to five) can typically undergo the consenting and enrolment evaluation process daily at each site. Subjects are not screened if they are participating in a different adherence study, which occurs at one clinic site. To determine the comparative characteristics of study participants with the general clinic population, we will compare baseline characteristics of a sample of non-study participants with characteristics of subjects enrolled in our study. Since mobile phone access is an enrolment criterion, we will include comparison of baseline characteristics of study enrolees with other clinic attendees who do not have phone access.

#### Primary outcomes

The first primary outcome is patient adherence to HAART at twelve months. (See Appendix) This is assessed by self-report using a standard questionnaire of the number of pills missed in the last 30 days and calculating percent adherence based on the expected number of doses taken. Percent adherence will be analyzed as a continuous variable and dichotomous variable. Subjects are considered either 'adherent' or 'non-adherent' using an optimal adherence cut off of > 95% of medications taken as directed.[1] Adherence is assessed at each follow-up visit and 'ever-non-adherent' will be our dichotomous outcome. The twelve month scheduled follow-up (defined, allowing for non-exact timing of clinic attendance, as follow-up up to 15 months if patients not present at 12 months) is the primary analysis. The six month assessment will allow for evaluating trends but is not the primary outcome.

The second primary outcome is suppression of plasma HIV RNA load at twelve months after initiating ART. If patients are adherent to HAART regimens, absorb the drugs normally, and if their virus is not resistant to the HAART drugs in the regimen, then plasma HIV load should be suppressed to below detectable levels (≤400 copies per ml) by six months and remain suppressed thereafter. Due to variability in when patients show up for scheduled appointments, we accept follow-up on or after 20 weeks as the six month outcome visit. Analysis of 'suppressed' (≤400 copies/ml) versus 'failure' to suppress (>400 copies/ml) at twelve months as a dichotomous variable is the primary analysis. However, decrease in plasma HIV load copies from baseline to twelve month follow-up will also be analyzed as the difference in a continuous variable from baseline to twelve months.

#### Secondary outcomes

The potential benefits of the intervention are quite broad, so multiple secondary outcomes are of interest and were selected through academic collaboration. The secondary outcomes fall under the categories of health, quality of life, social factors, and economic indicators (see Table 1). The primary purpose of taking antiretroviral medications for HIV infection is to maintain or restore health in someone who is HIV infected. Several other factors that contribute to a subject's prosperity or overall well-being may be affected directly (by way of enhanced support mechanisms associated with the intervention), or indirectly (through improvements in health related to HAART effect). We therefore feel it is important to assess each of these outcomes independently.

**Table 1:** Secondary Outcome Measures

**Secondary outcome measures (6 months and 12 months post initiation of HAART)**	**Type**
*Health outcomes*	
Self-reported adherence as a percentage	Binary
Suppressed HIV viral load (in copies)	Continuous
Immune reconstitution (change in CD4 T cell count from baseline)	
Time to virological failure	Continuous
Weight gain [lbs] and BMI	Time-to-event
Occurrence of opportunistic infections (OIs)	Continuous
Time to reporting of adverse drug events (ADEs)	Binary
Deaths (all cause)	Time-to-event
*Quality of life (QOL)*	Time-to-event
SF-12	
Satisfaction with care provided	Continuous
*Social factors*	Continuous
Level of disclosure of HIV status	
Impression of stigma	Binary
Family dyamics	Continuous
*Economic factors*	Continuous
Employment attendance	
Household member school attendance	Continuous
Cell phones lost/stolen	Continuous

HAART = highly active antiretroviral therapy, SF-12 = short form 12 adapted for regional application in Kiswahili.

In addition, we hypothesize that the intervention will lead to improved retention on ART.[5] Because participants are actively traced for twelve month follow-up visits, we will use composite retention outcomes. We define 'retained' as someone who followed-up on their scheduled visit without being traced and is still taking ART versus 'unretained' which includes subjects who stopped taking ART before their twelve month visit, who are lost to follow-up, or required active tracing after missing their scheduled twelve month appointment. We will report each element of the composite endpoints.[6]

### Sample size

The primary objectives of this trial to improve adherence to HAART and suppression of HIV viral load evaluated at 12 months. The sample size calculation is based on the comparison between proportions of patients with adherence rates (measured as percent of adherence >95%) in the two groups at 12 months because this is the variable that required the larger sample size. The criterion for significance (alpha) has been set at 0.05. The test is 2-tailed, which means that an effect in either direction will be interpreted.

The sample was calculated using (Cary, NC). With the proposed sample size of 247 and 247 for the two groups (ie assuming a 1:1 allocation ratio), the study will have power of 80% to yield a statistically significant result using a chi-squared test (assuming an intention-to-treat principle for the analysis) at alpha = 0.05/2 (ie using the Bonferroni correction factor for the two primary outcomes).

This computation assumes 10% improvement in 'perfect adherence' over a baseline of 75% determined from rates in a large adherence trial by AMPATH in Western Kenya which we felt is the most representative data available. [7] This difference of 10% was selected as the smallest difference that would be important to detect, in the sense that any smaller difference would not be of clinical or substantive importance. It is also assumed that this difference is reasonable, in the sense that an effect of this magnitude could be anticipated in this field of research.

Assuming attrition rates of approximately 10% we intend to over-enrol by 10% resulting in an enrolment target of at least 534 participants for randomization (267 in the Intervention, and 267 in the Control).

### Data collection

Data collection tools include a baseline questionnaire, follow-up questionnaire (used at 6 and 12 month visits), the SF-12 quality of life assessment adapted for local content, GPS co-ordinates of the clinics and participants homes, and a household roster of economic indicators. Questionnaires include continuous and categorical variables and Likert scales. All questionnaires were designed in English and translated to Kiswahili then back translated to verify meaning. Questionnaires are administered by clinic staff fluent in the languages of participants. In instances where study participants speak only local languages, the clinician translates questions directly. Antiretroviral adherence is assessed at each follow-up visit by inquiring about missed doses in the previous 30 days and calculating percent adherence according to pill counts. A visual analogue scale is used to confirm calculated adherence rates. A weekly study log is kept to record the weekly SMS messages and responses including details related to phone access, medication adherence and health or illness issues as they arose. Telephone communications with subjects in the control arm are also recorded in a similar study log, even though they are not sent the weekly SMS messages. Clinic charts are reviewed for WHO stages and clinical data on health parameters and events. A study register is kept to record follow-up.

Attempts are made to obtain lab data from samples obtained at usual clinical care time points and thus are either collected at the same visit as the study questionnaires are administered or within two weeks if not the same day. The CD4 T cell counts are run on a FACScan (Becton Dickson) at a centralized site. Plasmas for HIV viral load testing (Roche Amplicor) are separated at the central laboratory for the Nairobi clinics, but separated and frozen at -20C and batched for transport from the rural district clinic. All plasmas are frozen at -80C at the central lab. Viral loads are run in batches using manufacturer controls.

### Analysis plan

#### Analysis of primary and secondary outcomes

The trial will be reported according to the CONSORT standards for reporting randomized trials. We will use intention to treat (ITT) principles for primary outcome analysis and conduct additional sensitivity and 'per protocol' analysis separately as outlined below. Intention to treat implies all subjects randomized are considered in outcomes as per randomization. In this analysis we will impute missing outcome data as treatment failures. For adherence assessment, missing data at 12 months will be considered as 'non-adherent'; for viral suppression analysis, missing data at 12 months will be considered as virologic failures. We will use multiple imputation for imputing missing data that are not directly related to adherence. It is considered the gold standard for handling missing data because it leads to unbiased estimates of the effect and standard errors compared to single imputation methods.[8]

#### Sensitivity Analyses

Sensitivity analysis and per-protocol analysis includes study subjects who meet certain criteria. For sensitivity, additional analysis of outcomes as stated above will include only subjects who completed follow-up at the primary 12 month endpoint. Because the SMS intervention protocol requires active participation of study subjects to respond and thus conduct the intervention as fully intended, per-protocol analyses will also be performed. Study participants in the intervention arm who respond to the weekly SMS's with an SMS back (active response) at least 80% of the time with the 48 hour time frame will be considered highly protocol-adherent; those who respond 50% to 80% of the time will be considered moderately protocol-adherent; those who respond less than 50% will be considered poorly protocol-adherent; and those that respond less than 20% will be considered least protocol-adherent. Subjects who are randomized to the SMS intervention but never respond with a return SMS, for whatever reason, will be considered as never responding and not included in the sensitivity analysis. We will also perform a sensitivity analysis to assess the impact of potential clustering of patients cared for by the same clinic.

#### Sub-group analyses

Because a large clinical trial on this type of intervention has never been done before, we are interested in the interventions effect on various subgroups and the effect of multiple covariates on treatment outcomes. Subgroups of particular interests and potential covariates proposed are outlined in Table 2. Treatment arms will be compared with respect to potential covariates using continuous and categorical univariate analyses.

**Table 2:** List of subgroups and potential covariates

**Subgroups of interest**	**Potential covariates**
Urban vs. rural	Age
By clinic	Gender
Female vs. male	Distance from clinic
Phone ownership (owned vs. shared)	Income level
Occupational designation	Cost to attend clinic
Level of education	Level of literacy
Usual mode of transportation used	Baseline quality of life
	HIV staging
	Level of disclosure of status
	Crossover into intervention

HAART = highly active antiretroviral therapy.

#### Statistical methods

The intervention arm (SMS) will be compared against the control (SOC) for all primary analysis. We will use chi-squared test for binary outcomes, and T-test for continuous outcomes. For subgroup analyses, we will use regression methods with appropriate interaction terms (respective subgroup × treatment group). Multivariable analyses will be based on logistic regression (See Table 2) for binary outcomes and linear regression for continuous outcomes. We will examine the residual to assess model assumptions and goodness-of-fit. For timed endpoints such as mortality we will use the Kaplan-Meier survival analysis followed by multivariable Cox proportional hazards model for adjusting for baseline variables. We will calculate Relative Risk (RR) and RR Reductions (RRR) with corresponding 95% confidence intervals to compare dichotomous variables, and difference in means will be used for additional analysis of continuous variables. P-values will be reported to four decimal places with p-values less than 0.001 reported as p < 0.001. Up-to-date versions of SAS (Cary, NC) and SPSS (Chicago, IL) will be used to conduct analyses. For all tests, we will use 2-sided p-values with alpha = < 0.05 level of significance. We will use the Bonferroni method to appropriately adjust the overall level of significance for multiple primary outcomes, and secondary outcomes.

To assess the impact of potential clustering for patients cared by the same clinic, we will use generalized estimating equations [GEE] (ref) assuming an exchangeable correlation structure. Table 3 provides a summary of methods of analysis for each variable. Professional academic statisticians (LT, RN) blinded to study groups will conduct all analyses.

**Table 3:** Variables, Measures and Methods of Analysis

**Variable/Outcome**	**Hypothesis**	**Outcome Measure**	**Methods of Analysis**
1) **Primary**	Intervention improved outcome from baseline to 6 months		
			
a) Adherence at 12 months		Percent adherence in previous 30 days >95% [binary]	Chi-squared test
			
b) Suppression of HIV viral load at 12 months		Viral load ≤400 copies/ml [binary]	Chi-squared test

2) **Secondary** Adherence percentage at 12 months	improvement occurred	Adherence % (>95%) [binary]	Chi-squared test

HIV viral load at 12 months	improvement occurred	Viral load (copies)	T-test

Immune reconstitution (change in CD4 T cell count from baseline)	improvement occurred	Cd4 T-cells/mm^3 ^(continuous)	T-test

Time to virological failure	Improvement occurred	Virological failure after successful suppression	Kaplan-Meier survival analysis

Weight gain [lbs] and BMI	improvement occurred	Change in weight (lbs) and BMI [continuous]	T-test

Occurrence of opportunistic infections (OIs)	improvement occurred	Presence of AIDS defining opportunistic infection [binary]	Chi-squared test

Time to reporting of adverse drug events (ADEs)	improvement occurred	Presence of drug-related adverse event [time to event]	Kaplan-Meier survival analysis

Deaths (all cause)	improvement occurred	All-cause mortality [binary]	Chi-squared test and Kaplan-Meier survival analysis

SF-12	improvement occurred	Quality pf life questionnaire [continuous]	T-test

Satisfaction with care provided	improvement occurred	Questionnaire [continuous]	T-test

Level of disclosure of HIV status	improvement occurred	Disclosed to a family member [binary]	Chi-squared test

Impression of stigma	improvement occurred	Questionnaire [continuous]	T-test

Family dyamics	improvement occurred	Questionnaire [continuous]	T-test

Employment attendance	improvement occurred	Questionnaire [continuous]	T-test

Household member school attendance	improvement occurred	Questionnaire [continuous]	T-test

Cell phones lost/stolen	improvement occurred	Presence of cellphone [binary]	Poisson regression

Stopped taking HAART	improvement occurred	Self-report [binary]	Chi-squared test

Required active tracing for 12 month follow-up	improvement occurred	Field officers [binary]	Chi-squared test

3) **Subgroup Analyses:**			Regression methods with appropriate interaction term
Urban vs. rural	Distance affects adherence		
Female vs. male	Sex affects adherence		
Phone ownership (owned vs. shared)	Ownership affects adherence		
Level of education	Low education affects adherence		

4) **Sensitivity Analyses:**	improvement occurred	All outcomes	
a) Per protocol analysis			a) Chi-squared/T-test test
b) Adjusting for baseline covariates			b) Multivariable regression
c) clustering among individuals within a clinic			c) GEE

IMPORTANT REMARKS:

• The GEE[11] is a technique that allows to specify the correlation structure between patients within a hospital and this approach produces unbiased estimates under the assumption that missing observations will be missing at random. An amended approach of weighted GEE will be employed if missingness is found not to be at random.[12]

• In all analyses results will be expressed as coefficient, standard errors, corresponding 95% and associated p-values.

• Goodness-of-fit will be assessed by examining the residuals for model assumptions and chi-squared test of goodness-of-fit.

Bonferroni method will be used to adjust the overall level of significance for multiple secondary outcomes.

### Nested studies

#### Descriptors from the study logs

It is unknown what types of problems and issues will arise as a result of the cell phone intervention initiated by the study protocol. Detailed study logs kept by the study clinicians will allow description of the issues raised by patients during phone contact and how they were handled by the clinicians in both the SMS intervention group and standard of care control group.

#### Healthcare worker surveys

The nurses and clinical officers (clinicians) that manage the SMS intervention will be asked to complete self-administered questionnaires regarding their experiences. They will also be asked about their time commitments and work load before and after instituting the intervention.

#### Qualitative interviews

Semi-structured interviews will be designed to investigate in-depth experiences of subjects enrolled in the study. Twenty participants are targeted for a balance of gender and other characteristics such as occupation and lifestyle. Interviews are conducted in English or KiSwahili and in the latter case back-translated into English by two separated translators.

### Ethics

The study is being conducted in accordance with the Helsinki declaration and established guideline for research on human subjects. The study protocol was approved by the University of Manitoba and Kenyatta National Hospital ethics review boards. All participants provide written consent. This study does not have a Data and Safety Monitoring Board. We do not have a code-breaking procedure for randomization as the study is unblinded, except for analysis. All protocol amendments will be approved first by the study primary investigators (RTL, JK, LG, and FP). The investigators have no competing interests. We will not provide post-trial care using SMS until approval from the ART funding body. All study results will be publicly available.

## Discussion

The intervention being tested provides the opportunity to improve health care delivery in resource limited settings. Our study is one of several planned randomized trials evaluating cell-phone based technology for adherence. In other settings, Collier et al. demonstrated that an intensive regular telephone-based adherence counselling intervention did not significantly improve adherence to HAART in the United States, Italy, and Puerto Rico[8]. In a separate randomized trial in the United States, a structured telephone counselling intervention did improve adherence to HAART and there was a trend toward improved clinical outcomes[9]. A recently piloted SMS text reminder to improve adherence to ART among Los Angeles youths was acceptable and showed early benefit on adherence [10]. In another study, a structured cell phone intervention to help HIV patients attending an inner-city clinic in Texas quit smoking was highly successful[10]. We feel that the benefits of mobile telephony in resource limited settings may be greater than in countries with developed economies, since they are more widely used in management of daily life activities, and mark a great contrast to pre-mobile phone era communication infrastructures. Additionally, our study is unique in that it focuses on a simple structured SMS protocol without a specified counselling intervention. Thus we are directly testing the ability for improved, efficient communications to have an impact on patient care.

The multi-site randomized controlled trial design was specifically designed to incorporate a broad sampling of populations attending ART care clinics in Kenya. This approach risks diluting the statistical power to detect treatment responses which could be more easily seen in more homogenous patient groups. However, we feel there is a common thread of impact of cell phone usage across all populations in the region. This study design and analysis plan will allow us to evaluate several factors that may contribute a variable array of patient benefits. We are also aware that by consenting and participating in this study, both the healthcare providers and study subjects (including controls), are made more aware of the potential benefits of increased cell phone communications even outside the SMS protocol. This is known as the Hawthorne effect of participation in clinical trials, and may reduce the power to compare the effect of the SMS intervention against the true standard of care seen in non-study environments. Since it would be unethical to restrict communications between health care providers and patients, we have applied for ethics approval to compare adherence and other outcomes in this clinical trial with non-trial participants at the same or similar clinics. We recognize that blinding participants in a trial such as ours is impossible. However, our SMS texts are delivered in such a way as to a) reduce likelihood of disclosure to others around the patient and b) to act as a reminder of health, rather than HIV status, as our texts simply state 'how are you?'

All factors contributing to the success or failure of the intervention to improve health care management of patients are unlikely to have been predicted *a priori*. Therefore the results of this trial, including the qualitative nested studies and experiences of participating clinicians, will not only test the effectiveness of the described intervention protocol, but will instruct further development of the use of mobile telephony to improve health management in resource limited settings.

## Competing interests

The authors declare that they have no competing interests.

## Authors' contributions

RTL conceived of the study. AK, EN, SB, PR, WJ, JH, and MC initiated the study design and JK and LG helped with implementation. RTL, JK, LG, and FP are grant holders. LT and EM provided statistical expertise in clinical trial design and RN is conducting the primary statistical analysis. All authors contributed to refinement of the study protocol and approved the final manuscript.

## Appendix

### Primary Outcome Measures

HAART = highly active antiretroviral therapy.

Primary binary outcomes (12 months post initiation of HAART)

1. Self-reported adherence (>95%) in previous 30 days)

2. Suppressed HIV viral load (≤400 copies/ml)
